# Comparison Study of Two Differently Clicked ^18^F-Folates—Lipophilicity Plays a Key Role

**DOI:** 10.3390/ph11010030

**Published:** 2018-03-17

**Authors:** Kathrin Kettenbach, Laura M. Reffert, Hanno Schieferstein, Stefanie Pektor, Raphael Eckert, Matthias Miederer, Frank Rösch, Tobias L. Ross

**Affiliations:** 1 Johannes Gutenberg-University Mainz, Institute of Nuclear Chemistry, Fritz-Straßmann-Weg 2, 55128 Mainz, Germany; kathrinkettenbach@web.de (K.K.); hanno.schieferstein@gmx.de (H.S.), raphael_eckert@web.de (R.E.), frank.roesch@uni-mainz.de (F.R.); 2 Hannover Medical School, Department of Nuclear Medicine, Radiopharmaceutical Chemistry, Carl-Neuberg-Str. 1, 30625 Hannover, Germany; reffert.laura@mh-hannover.de; 3 University Medical Center of Johannes Gutenberg-University Mainz, Polyclinic of Nuclear Medicine, Langenbeckstr. 1, 55131 Mainz, Germany; stefanie.pektor@unimedizin-mainz.de (S.P.), matthias.miederer@unimedizin-mainz.de (M.M.)

**Keywords:** ^18^F-folates, PET, folic acid, folate receptor, click chemistry, copper-catalyzed click, strain promoted click

## Abstract

Within the last decade, several folate-based radiopharmaceuticals for Single Photon Emission Computed Tomography (SPECT) and Positron Emission Tomography (PET) have been evaluated; however, there is still a lack of suitable ^18^F-folates for clinical PET imaging. Herein, we report the synthesis and evaluation of two novel ^18^F-folates employing strain-promoted and copper-catalyzed click chemistry. Furthermore, the influence of both click-methods on lipophilicity and pharmacokinetics of the ^18^F-folates was investigated. ^18^F-Ala-folate and ^18^F-DBCO-folate were both stable in human serum albumin. In vitro studies proved their high affinity to the folate receptor (FR). The lipophilic character of the strain-promoted clicked ^18^F-DBCO-folate (logD = 0.6) contributed to a higher non-specific binding in cell internalization studies. In the following in vivo PET imaging studies, FR-positive tumors could not be visualized in a maximum intensity projection images. Compared with ^18^F-DBCO-folate, ^18^F-Ala-folate (logD = −1.4), synthesized by the copper-catalyzed click reaction, exhibited reduced lipophilicity, and as a result an improved in vivo performance and a clear-cut visualization of FR-positive tumors. In view of high radiochemical yield, radiochemical purity and favorable pharmacokinetics, ^18^F-Ala-folate is expected to be a promising candidate for FR-PET imaging.

## 1. Introduction

Folic acid (vitamin B9) is an essential nutrient for de novo DNA synthesis [1,2]. For naturally occurring folic acid as well as its derivatives the generic term folate is used. To ensure sufficient folate supply different transport mechanisms are available. Circulating folates in the blood stream are carried via transporters across the cellular membrane: the anionic reduced folate carrier (RFC) transports tetrahydrofolate (THF) and the proton-coupled folate transporter (PCFT) various reduced folates [3,4]. In contrast, oxidized folates are transferred via the folate receptor (FR)-mediated endocytosis into cells. The FR has a high affinity for folic acid (K_d_ of 1 nM) and is overexpressed on a variety of highly proliferating cancer cells [5,6]. However, in healthy tissues the FR expression is strictly limited to a few sites such as the kidneys (proximal tubule), choroid plexus, lung, salivary glands and the placenta, making it an ideal oncological target for imaging and therapy [5,7,8].

Within the past ten years, several ^18^F-folate derivatives have been developed using either ^18^F-labeled prosthetic groups [9,10,11,12,13] or direct labeling strategies [14,15]. In 2008, the first ^18^F-click folate was reported, resulting in excellent overall yields of up to 35%, but due to the lipophilic character the in vivo behavior was unfavorable [9]. To decrease lipophilicity, an ^18^F-click folate with an oligoethylene glycol spacer was developed by our group [12]. This radiotracer showed significantly reduced hepatobiliary excretion, while maintaining tumor uptake. Further approaches provided directly labeled ^18^F-folate derivatives with high affinities to the folate receptor (K_i_ = 1.8 ± 0.1 mM), but radiochemical yields for its synthesis were less than 9% [15]. Attaching an albumin-binding moiety to the ^18^F-folate radiotracer enhanced blood circulation time and an increased tumor-to kidney-ratio (0.88 ± 0.12 compared to 0.23 ± 0.04% ID/g) [16].

As these examples show, the major goal in ^18^F-folates research is to achieve the right balance between pharmacokinetics (reduced abdominal background) and radiochemistry. This aim was first achieved by Fischer et al. in 2012 by using a clickable ^18^F-FDG derivative as a polar ^18^F-prosthetic group, which was attached to a folate derivative via copper-catalyzed azide-alkyne click cycloaddition (CuAAC). They obtained for the first time a good radiochemical yield (RCY) of 25% and a high uptake of 10% ID/g tissue in a FR-positive tumor. However, the long preparation time of about 180 min can be seen as a drawback [10]. The fact that radiolabeled amino acids show great potential as clinically used radiotracers and that most relevant biomolecules (i.e., peptides, antibodies and proteins) are based on amino acids, encouraged us to develop a novel clickable ^18^F-prosthetic group based on alanine (^18^F-Alakyne) [17]. ^18^F-labeled folate derivatives are great examples where click-prosthetic groups usually impair pharmacokinetics [9,10,11,12]. One disadvantage of these click reactions is the need of a cytotoxic copper catalyst. Therefore, a multitude of strained ^18^F-prosthetic groups have been developed, which can be attached without the need of a copper species (strain-promoted azide alkyne cycloaddition, SPAAC). Recently, a new ^18^F-prosthetic group based on dibenzocyclooctyne (^18^F-DBCO) has been developed in our group and applied for radiolabeling of a folate derivative (^18^F-DBCO-folate) among other biomolecules [18]. The aim of this work was the comparison of a ^18^F-folate synthesized via copper-free, using the known ^18^F-DBCO-folate, with a novel ^18^F-folate, ^18^F-Ala-folate, synthesized via copper-catalyzed click reaction regarding their in vitro characteristics and in vivo performance in a KB-xenograft bearing mouse model.

## 2. Results 

### 2.1. Organic Chemistry

**Scheme 1:**
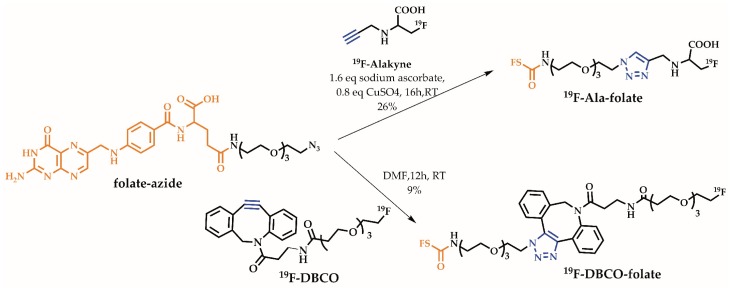
Synthesis of both reference compounds ^19^F-Ala-folate and ^19^F-DBCO-folate starting with folate-azide.

Both non-radioactive reference compounds of the prosthetic groups, ^19^F-Alakyne and ^19^F-DBCO as well as their labeling precursors, were synthesized as previously reported [17,18]. The two prosthetic groups were clicked to an azido-functionalized folate (folate-azide), which was prepared by amide coupling of diacetyl protected pteroic acid and an azido-functionalized glutamate following a published procedure with minor changes [12] (see supporting information Scheme S1.2). Instead of using N^2^-N,N-dimethylaminomethylene-10-formylpteroic acid, N^2^,N^10^-diacetyl pteroic acid was utilized which was synthesized by stepwise degradation of folic acid as described elsewhere [19] (see supporting information Scheme S1.1). As depicted in Scheme 1, the folate-azide was either clicked via copper-free SPAAC to ^19^F-DBCO to obtain ^19^F-DBCO-folate or via CuAAC to ^19^F-Alakyne yielding ^19^F-Ala-folate. 

### 2.2. Radiochemistry

^18^F-DBCO-folate was prepared in 120 min according to the published procedure [18] yielding a radiochemical yield (RCY) of 3.2 ± 1.8% (≥95% radiochemical purity, for HPLC chromatograms see supporting information Figure S1). Even after HPLC and C18 purification an effective separation from non-radioactive precursor was not achievable, resulting in a high load of non-radioactive material (apparent molar specific activity: 2.1 ± 0.3 GBq/µmol).

**Scheme 2:**
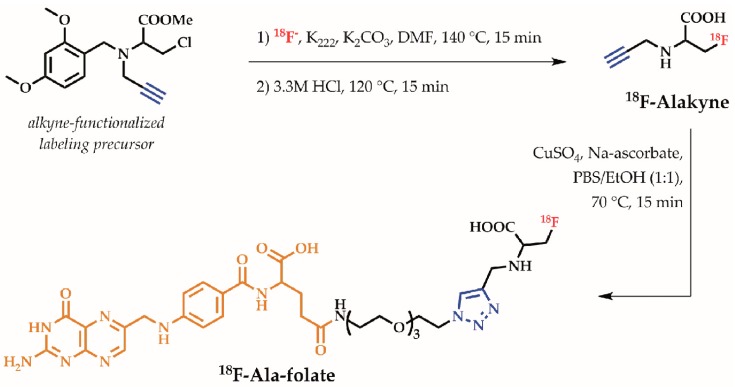
Synthesis of ^18^F-Alakyne and subsequent conversion to ^18^F-Ala-folate.

With some minor changes the radiolabeling of ^18^F-Alakyne was adapted from literature as displayed in Scheme 2 [17]. Instead of using a microwave, conventional heating was used resulting in RCY of 29.4 ± 7.7% (for HPLC chromatogram see supporting information Figure S2). Subsequently, ^18^F-Alakyne was clicked to folate-azide with an almost quantitative conversion within 15 min, yielding ^18^F-Ala-folate in an overall RCY of 19.3 ± 2.8% after HPLC purification (≥97% purity, for HPLC chromatograms see supporting information Figure S3). However, as a complete separation from the non-radioactive chloro-precursor of the ^18^F-Alakyne was not achieved, also the chloro-precursor clicked to the folate-azide, resulting in a high load of non-radioactive material in the final product. As described previously, synthesis of a tosylate precursor could not be achieved due to its instability [17]. Consequently, the (apparent) specific activity of ^18^F-Ala-folate was as low as for the prosthetic group (2.3 ± 0.3 GBq/µmol). Good RCYs of up to 22% within 150 min synthesis time could be obtained. 

### 2.3. Lipophilicity

The lipophilicity was determined by shake flask method in an octanol/PBS mixture. Table 1 gives an overview of logD values for ^18^F-Alakyne and ^18^F-Ala-folate as well as for ^18^F-DBCO and ^18^F-DBCO-folate. Connecting folic acid to both prosthetic groups resulted in a drop in logD values. As expected, presence of the lipophilic, bulky DBCO group resulted in the highest logD value for the ^18^F-DBCO-folate. In contrast, ^18^F-Ala-folate has a more hydrophilic character derived from the high hydrophilicity of both folic acid and ^18^F-Alakyne. The reduced lipophilicity is expected to be an indicator for less non-specific background signals and a higher renal clearance rate. 

The relative lipophilicity (k′ value) can be used to compare different radiofolates based on their retention in the RP-HPLC as shown in Table 2. ^19^F-Ala-folate has a slightly lower k′ value than native folic acid demonstrating the negligible influence of ^19^F-Alakyne as prosthetic group on the lipophilicity. Considering the bulky hydrophobic DBCO group the change in k′ value is not surprising. 

## 3. In Vitro Studies

### 3.1. Stability Studies in Human Serum Albumin 

The in vitro stability was assessed in human serum albumin at 37 °C. After 1 h and 2 h incubation very low metabolic degradation was observed for both radiofolates proving the high metabolic stability (see supporting information for diagram, Figure S4). After 2 h incubation >90% was still intact for ^18^F-DBCO-folate and >85% for ^18^F-Ala-folate, indicating no considerable stability issues for the duration of 60–90 min µPET scans. 

### 3.2. Binding Affinity Studies

**Figure 1:**
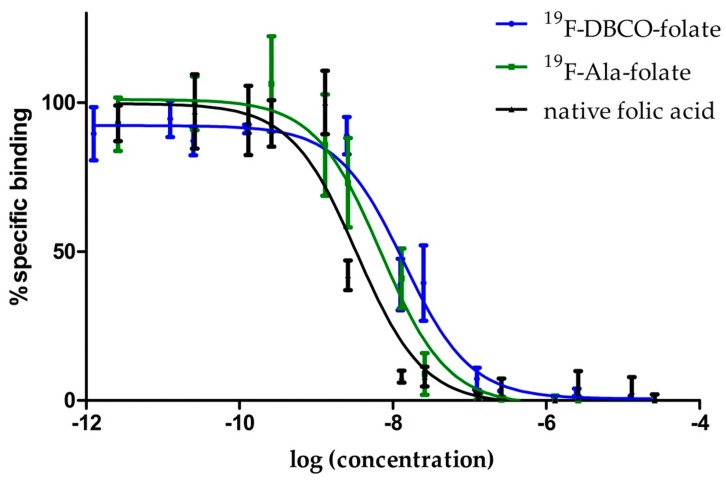
Displacement assay: % specific bound [^3^H]folic acid against ^19^F-DBCO-folate and ^19^F-Ala-folate.

Figure 1 shows the displacement curve of [^3^H]folic acid (8 × 10^−7^ M) with ^19^F-DBCO-folate and ^19^F-Ala-folate in various concentrations (10^−4^ to 10^−12^ M) to determine the inhibitory concentration IC_50_ and the assay independent K_i_ value. For both new radiofolates, the IC_50_-values are in a comparable nanomolar range, indicating an excellent affinity for targeting the folate receptor.

The pIC_50_ value displays the negative logarithm of IC_50_ and eases the comparison between varying compounds and their IC_50_-values. Table 3 provides an overview of IC_50_, pIC_50_ and K_i_ values for native folic acid and both new ^19^F-folate derivatives. All three have similar pIC_50_ values which implicates the minor influence of derivatization on receptor recognition. These data demonstrate again that the folate receptor tolerates a variety of changes in the non-pharmacophore part, the glutamic acid moiety, and even bulky groups such as boron cluster are recognized with no significant loss in binding affinity [20].

### 3.3. Internalization Studies

**Figure 2:**
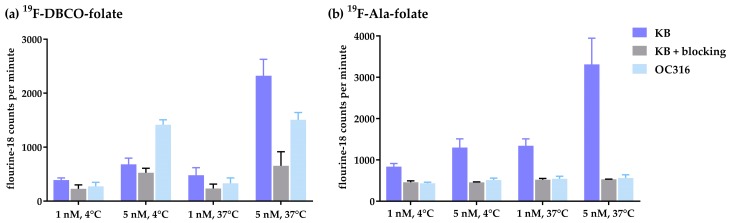
Internalization of ^18^F-DBCO-folate (**a**) and ^18^F-Ala-folate (**b**) in KB and OC316 cells at 4 °C and 37 °C for 1 h including blocking with native folic acid.

Both ^18^F-folate radiotracer ^18^F-DBCO-folate and ^18^F-Ala-folate were tested using FR-positive KB cells and FR-negative OC316 cells including blocking studies with an excess of native folic acid. Two different temperatures (4 °C for receptor binding, 37 °C for receptor binding and internalization) and two different concentrations were assessed. Figure 2 displays the results of internalization studies with ^18^F-DBCO-folate and ^18^F-Ala-folate. Both radiofolates show a concentration as well as temperature depended increase of activity in the FR positive cells. Due to the lipophilic character of ^18^F-DBCO-folate, a distinct non-specific binding was detected for blocked KB cells and especially for the FR-negative OC316 cells. Surprisingly, the uptake at 4 °C and 5nM in OC316 cells was much higher than that seen in KB cells. This effect is not fully understood, but we presume the high lipophilicity in combination with the increased concentration being responsible for this high non-specific binding. The distribution of ^18^F-DBCO-folate at 5 nM and 37 °C shows that only about 3% of the tracer was actually internalized and about 7% were receptor bound. Thus, about 90% of ^18^F-DBCO-folate remained in solution, which is ideal for such a radiotracer assay set-up (see supporting information for pie chart of activity distribution, Figure S6). For ^18^F-Ala-folate the overall receptor-bound and internalized fractions are similar to ^18^F-DBCO-folate (see supporting information for pie chart of activity distribution, Figure S7). As expected for the more hydrophilic ^18^F-Ala-folate a lower non-specific binding was observed. A clearly visible blocking effect using native folic acid underlines the specificity in FR-positive KB cells. 

## 4. Animal Studies

### 4.1. Ex Vivo Biodistribution Studies 

**Figure 3:**
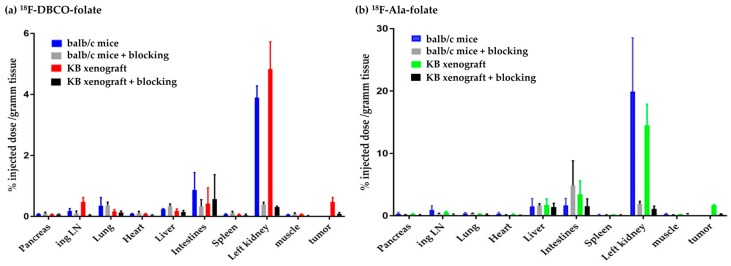
Results of biodistribution of ^18^F-DBCO-folate (**a**) and ^18^F-Ala-folate (**b**) at 60 min p.i.

The results of the biodistribution studies in healthy (*n* = 5) and KB-tumor bearing mice of ^18^F-DBCO-folate (*n* = 6) and ^18^F-Ala-folate (*n* = 10) are shown in Figure 3 (see supporting information for values in table form, Table 1 and Table 2). Furthermore, FR specificity was demonstrated by preinjection of excess native folic acid for blocking.

The highest uptake for both radiofolates was found in the urine confirming a pronounced renal clearance. Radioactivity in blood was low after 60 min p.i. (^18^F-DBCO-folate: 0.09 ± 0.04% ID/g tissue, ^18^F-Ala-folate: 0.17 ± 0.05% ID/g tissue) indicating a fast clearance of the blood pool. Highly specific uptake was found in the FR-positive kidneys, as greater than 93% of the uptake was reduced for both radiofolates in the blocked groups. Non-specific accumulation was found for both in liver and the gastrointestinal tract. In general, the ^18^F-Ala-folate showed higher % ID/g values in all tissues. In comparing the tumor uptake of ^18^F-DBCO-folate with ^18^F-Ala-folate, a 3-times higher uptake (1.68 ± 0.13% ID/g tissue) was observed for ^18^F^-^Ala-folate. In the blocked group 81% less activity in the tumor for ^18^F-DBCO-folate and 85% reduction for ^18^F-Ala-folate were found, indicating a specific FR-mediated uptake. The logD values are reflected in the in vivo results: the more lipophilic ^18^F-DBCO-folate shows a higher non-specific background than the ^18^F-Ala-folate which is also depicted by the lower tumor to organs ratios in Table 4. The tumor-to-blood-ratio doubled for the ^18^F-Ala-folate in comparison to the ^18^F-DBCO-folate, which we assume is due to the lipophilic character of the DBCO-moiety. In addition, the tumor-to-kidney and the tumor-to-muscle ratios are higher for the ^18^F-Ala-folate.

### 4.2. In Vivo PET Studies

Healthy and KB tumor-bearing mice were investigated in µPET imaging studies. First, the pharmacokinetic profiles of the radiotracers were obtained in 60 min dynamic scans in KB tumor-bearing mice. These scans revealed a favorable clearance of ^18^F-DBCO-folate (*n* = 8) and ^18^F-Ala-folate (*n* = 12) from the blood pool and a rapid wash-out from non-target organs. The tumor uptake stayed constant over time, while the kidney accumulation decreased to the levels of receptor-specific uptake due to the renal clearance of the radiotracer (see supporting information for kinetics, Figure S8). Therefore, static scans were performed 50 min post injection (*n* = 6), to allow an efficient clearance of unbound tracer. 

**Figure 4:**
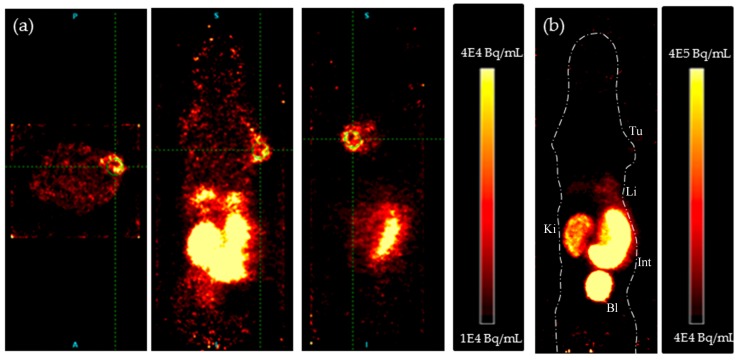
^18^F-DBCO-folate PET images of a KB-tumor bearing mouse (50–60 min p.i.). (**a**) representative transverse, coronal and sagittal plane with a green crosshair pointing at KB-tumor and (**b**) Maximum intensity projection. Tu = KB-tumor, Li = liver, Ki = kidney, Int = intestines, Bl = bladder.

As depicted in Figure 4b, visualization of KB tumors in the maximum intensity projection (MIP) PET images was impeded by the lipophilicity of ^18^F-DBCO-folate. This aligns with the results of *ex vivo* biodistribution, where the KB-tumor displayed low levels of activity (0.48 ± 0.14% ID/g). By setting thresholds in the sagittal, coronal, and transversal slices, the FR-positive tumors can be visualized. Injection of a blocking dose folic acid (*n* = 6) resulted in a decreased kidney signal demonstrating a specific uptake (see supporting information Figure S9a). Furthermore, a considerable amount of activity was found in the abdominal region, more precisely in the intestines due to the hepatobiliary excretion of ^18^F-DBCO-folate. Negligible uptake was observed in the bones demonstrating that ^18^F-DBCO-folate is stable against in vivo defluorination.

**Figure 5:**
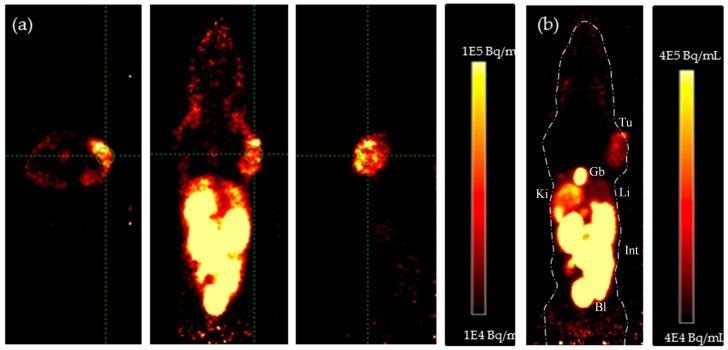
^18^F-Ala-folate. PET images of a KB-tumor bearing mouse. Static scan over 10 min 50 min p.i. (**a**) representative transverse, coronal and sagittal plane with a green crosshair pointing at KB-tumor and (**b**) Maximum intensity projection. Tu = KB-tumor, Gb = gallbladder, Li = liver, Ki = kidney, Int = intestines, Bl = bladder.

In contrast, the less lipophilic ^18^F-Ala-folate displayed the KB-tumor in the MIP (Figure 5b). Also, a good visualization of the kidney cortex proves folate receptor specific uptake of the ^18^F-Ala-folate in FR-positive tissues. Additionally, a non-specific abdominal accumulation is still observed, but the tumor-to-background contrast is higher compared to ^18^F-DBCO-folate. Blocking studies with native folic acid were performed to demonstrate again specific binding in kidneys and KB tumors, whereas activity in the gallbladder (non-specific uptake, excretion) was not reduced, or rather slightly reduced for the liver (see supporting information Figure S9b). 

## 5. Discussion

The aim of this work was to evaluate the influence of two clickable ^18^F-prosthetic groups coupled to folic acid. For the coupling, either copper catalyzed or strain promoted click chemistry was used and both folates were tested for their pharmacological properties. Click chemistry enables ^18^F-labeling without the utilization of protecting groups and is an ideal tool for prosthetic group labeling. While diverse ^18^F-folates have already been synthesized via CuAAC, it is the first time a copper-free clicked ^18^F-click-folate is evaluated in µPET imaging and ex vivo biodistribution studies. 

Lipophilicity plays a crucial role for the pharmacokinetic fate of a compound influencing its ADME (absorption, distribution, metabolism, elimination) profile. Lipophilic compounds are associated with a higher non-specific binding to plasma proteins and pronounced hepatobiliary excretion, while polar compound are eliminated by the renal excretion pathway [21]. An ideal radiofolate should have a fast radiolabeling with high RCY and RCP, show a moderate lipophilicity, bind with high affinity to the folate receptor and be metabolically stable with a favorable ADME profile. These parameters were tested for ^18^F-DBCO-folate and ^18^F-Ala-folate with various methods. 

Compared to previously reported ^18^F-click folate derivatives [9,10,12], high radiochemical yields of up to 22% for ^18^F-Ala-folate were achieved. An improvement of specific activity by removing unlabeled precursor would enhance the preclinical performance. A long radiosynthesis time is not preferable for clinical applications and could be reduced by automatization sparing transfer time of reaction mixtures and having all instruments next to each other. Compared to direct labeled folate-PEG_12_-NOTA-AL^18^F with a radiosynthesis time of 35 min, radiolabeling via prosthetic groups is in general more time consuming (3 h for [^18^F]FDG-folate, 2.5 h for ^18^ F-OEG-folate) [12,16]. 

Differences in molecular weight and the pKa of a compound influence the lipophilicity. Judging from folate derivatives in the literature [11,14,16,22] the lower logD of 1.43 ± 0.08 for ^18^F-Ala-folate is still too high for a favorable biodistribution pattern. While in vitro studies confirmed the high folate receptor affinity for both radiofolates, the bulky and lipophilic DBCO group has a significant impact at the in vivo behavior and impairs the tumor uptake in a murine model. The binding affinities were not significantly altered compared to native folic acid. These results line up with previous reported ^18^F-folates [9,22,23]. In vivo, the ^18^F-DBCO-folate showed a reduced uptake in the FR positive tumors plus an increased non-specific background. This might be due to the increased lipophilicity as already demonstrated for a ^18^F-labeled cyclooctyne-based peptide targeting the α_v_β_6_ integrin [24]. With a slightly improved distribution coefficient (logD), the influence of lipophilicity is still seen in the high accumulation of ^18^F-Ala-folate in intestines and liver/gallbladder. However, the tumor-to-liver (gallbladder) ratio of ^18^F-Ala-folate (0.98) is 10-fold higher than that reported for folate-NOTA-Al^18^F (0.1) [23], and comparable to the ratios reported for copper clicked [^18^F] FB-folate, [^18^F]FE-folate and [^18^F]FDG-folate [22], ranging from 0.85 to 1.35, respectively. Although in general, tumor-to-background contrast for ^18^F-Ala-folate is higher than that for ^18^F-DBCO-folate, the reduced tumor-to-liver contrast was unexpected. However, the kinetics of the hepatobiliary excretion of ^18^F-DBCO-folate was slightly faster than that of ^18^F-Ala-folate, and at 60 min p.i. most of the excreted activity has already accumulated in intestines/feces. In contrast, ^18^F-Ala-folate activity remained in the liver and especially in the gallbladder at 60 min p.i., resulting in the reduced tumor-to-liver (gallbladder) ratio. This effect is supported by the in vivo µPET images at 60 min p.i., where the gallbladder activity of the ^18^F-DBCO-folate cleared already into intestines and feces.4. Conclusions 

In summary, two radiofolates, ^18^F-Ala-folate and ^18^F-DBCO-folate, have been evaluated with respect to their FR in vivo imaging ability. In general, finer tuning regarding the ADME profile is needed because there is still a need for a fluorine-18 labeled folic acid derivative to utilize for FR PET imaging. On the other hand, the ^18^F-Ala-folate is a promising candidate for FR PET imaging in vivo allowing visualization of FR-positive tissue and being available in high radiochemical yields after a multi-step, but convenient radiosynthesis. Furthermore, the situation in humans clearly differs from the one in murine models and imaging quality may increase, even in the abdominal region. Taking everything into account, we consider the ^18^F-Ala-folate as suitable for clinical investigations and translation.

## 6. Materials and Methods 

### 6.1. Materials

Reagents and solvents were purchased from Acros Organics, Alfa Aesar, Fisher Scientific, Fluka, Merck, Sigma Aldrich and VWR and used without further purification. The diammonium salt of [3´,5´,7,9-^3^H] folic acid was purchased from Hartmann Analytics. Nuclear magnetic resonance spectra (^1^H and ^19^F) were recorded using an AC-300-Spectrometer (300-MTh-T-NMR-spectrometer AC 300, Bruker BioSpin GmbH, Rheinstetten, Germany) and for ^13^C-NMR a Bruker Advance II-400-Spectrometer (400 MHz). Reactions were monitored using thin layer chromatography (silica gel 60 F254, Merck KGaA, Darmstadt, Germany) or high-performance liquid chromatography (HPLC). Information about compound characteristics and the HPLC methods can be found in the Supplementary Materials.

### 6.2. Organic Chemistry 

^19^F-DBCO-folate (non-radioactive) was obtained after stirring ^19^F-DBCO (4.2 mg, 8.7 µmol) and folate-azide (3.4 mg, 5.3 µmol) in 1 mL of dry DMF for 12 h at RT. The crude mixture was diluted with water (1–3 mL) and in portions injected into the semi-preparative HPLC system for purification to yield 8.5% (0.5 mg, 0.45 µmol) of the desired product, ^19^F-DBCO-folate after lyophilization.

For the synthesis of ^19^F-Ala-Folate, ^19^F-Alakyne (20.2 mg, 0.078 mmol) was dissolved in 4 mL ethanol and then copper(II)sulfate (9.8 mg, 0.062 mmol) in 0.5 mL PBS, folate-azide (50 mg, 0.078 mmol) in 3.3 mL PBS and sodium ascorbate (24.8 mg, 0.125 mmol) in 0.5 mL PBS were added. The reaction mixture was stirred at RT for 16 h. The crude mixture was diluted with water (5–10 mL) and in portions injected into the semi-preparative HPLC for purification. After lyophilization of the product fraction, ^19^F-Ala-folate was obtained in yields of 26% (9.2 mg, 0,021 mmol).

### 6.3. Radiochemistry: Labeling with n.c.a. [^18^F]Fluoride 

The procedure to produce dried n.c.a. [^18^F]fluoride ([^18^F]*KF@K_2.2.2_*) is described in the supporting information. 

Specific activities (SA) were determined from HPLC calibration curves obtained from the areas (mAU) of the UV-signals from different concentrations of ^19^F-DBCO-folate and ^19^F-Ala-folate.

#### 6.3.1. ^18^F-Ala-Folate

Radiolabeling of ^18^F-Alakyne followed the published procedure with minor changes [17]. The dry [^18^F]fluoride-base mixture was dissolved in 0.25 mL anhydrous DMSO, transferred into a sealed reaction vial containing the alkyne labeling precursor (1.0 mg, 3.1 µmol) in 0.25 mL DMSO and heated for 15 min to 140 °C. This was followed by quenching with ammonium formate buffer (50 mM, 20 mL). A C18-cartridge was used to separate the protected ^18^F-intemediate from unreacted [^18^F]fluoride. The intermediate was eluted by acetonitrile (1.5 mL). The solvent was removed under reduced pressure and the intermediate was dissolved with hydrochloric acid solution (3.3 M, 500 µL) and heated at 120 °C for 15 min. After cooling, the reaction mixture was neutralized with sodium hydroxide solution (3.3 M, 500 µL) and purified by HPLC. ^18^F-Alakyne was dissolved in a PBS:ethanol mixture (1:1, 1 mL) and CuSO_4_ (20 µmol in 40 µL PBS) was added followed by folate-azide (0.5 mg, 0.6 µmol) and sodium ascorbate (100 µmol in 40 µL PBS). The reaction mixture was heated to 70 °C for 15 min and purified using HPLC. For lipophilicity and cell experiments ^18^F-Ala-folate was dissolved in PBS, for stability tests and in vivo application sterile sodium chloride solution was used.

#### 6.3.2. ^18^F-DBCO-Folate

Labeling of ^18^F-DBCO and subsequent click reaction to folate-azide was performed as described elsewhere [18]. After HPLC purification, the product fraction was diluted with water (20 mL) and passed through a solid-phase cartridge (Sep Pak Light tC18, Waters GmbH, Eschborn, Germany. The ^18^F-DBCO-folate retained in the cartridge was eluted with EtOH/water (1:1, 1 mL) into a reaction vessel and ethanol was removed using a helium stream at 95 °C within 15 min. Formulation was the same as for ^18^F-Ala-folate.

### 6.4. Determination of Lipophilicty 

The octanol-water (buffer) partition coefficient of ^18^F-Alakyne and ^18^F-Ala-folate was determined using the standard shake-flask method [18]. Relative lipophilicity (k’ values) was determined by HPLC with an isocratic acetonitrile-phosphoric acid buffer eluent system at pH 2.4 as described elsewhere [15]. 

### 6.5. In Vitro Studies

As folate-receptor positive control, KB cells (derived from human cervical carcinoma; obtained from German Collection of Microorganisms and Cell Cultures), and OC316 cells (derived from human ovarian adenocarcinoma) as negative control were used. For FACS analysis, data of both cell lines see supporting information Figure S5. The cells were cultured in folate deficient RPMI 1640 medium containing 10% FCS, 1% penicillin-streptomycin and HEPES buffer (10 mM, 5 mL) grown at 37 °C in a humidified atmosphere containing 5% CO_2_. To determine the IC_50_ and K_i_ values of both ^18^F-folate derivatives, displacement assays using KB cells and [^3^H] folic acid were performed as described elsewhere [20]. To determine the internalized fraction of both new ^18^F-folate derivatives, different concentrations (1 and 5 nM) of the labeled agents were incubated with one million KB or OC316 cells at 4 °C and 37 °C. For blocking experiments, native folic acid (100 µg in 100 µL PBS buffer) was added prior to adding the tracer. After 1h, the supernatant was removed, cells washed with PBS and with an acetate buffer (pH 3). The radioactivity of all fractions was measured using a γ-counter (PerkinElmer 2470 Wizard2, Perkin Elmer, Rodgau, Germany).

#### 6.5.1. Stability Studies in Human Serum Albumin

For testing the stability, 200 µL of ^18^F-DBCO-folate or ^18^F-Ala-folate (ca. 4 MBq) were incubated at 37 °C in 500 µL human serum albumin. After predetermined time points plasma proteins were precipitated with 600 µL ice-cold acetonitrile followed by centrifugation (10,000 rpm, 10 min). An aliquot of the supernatant was injected into an analytical radio-HPLC. 

### 6.6. In Vivo Studies

All animal experiments were approved by the local ethical committee (permission no. 23177-07/G15-1-033). Balb/c and balb/c nude (Balb/c AnNRj-Foxn1nu) mice were kept on a folate-deficient rodent diet to reduce their blood level of folate to one found in humans [25]. After 7 days of acclimatization, human KB tumor cells (2.5 x 10^6^ cells in PBS) were inoculated in the right shoulder of each balb/c nude mouse. All in vivo treatments and imaging experiments were done under isoflurane anesthesia. Eleven or twelve days after inoculation, animals were intravenously injected with ^18^F-DBCO-Folate and ^18^F-Ala-Folate (5–8 MBq, 100–200 µL) via a lateral tail vein. For blocking studies, mice were injected with native folic acid (100 µg in 100 µL PBS) 2 min prior to radiotracer injection. PET/MRI-studies were performed on a nanoScan PET/MRI (Mediso, Budapest, Hungary). First MRI scans (Material Map for co-registration of the PET scan; 3D; GRE-EXT, FOV; slice thickness: 0.6 mm; TE: 2 ms; TR: 15 ms; flip angle: 25 deg) were acquired and then either a dynamic scan over 60 min starting with injection of the radiotracer or a static scan for 10 min after 50 min p.i. were performed. All mice, which underwent ex vivo biodistribution, were scanned first. Afterwards co-registered PET/MR images were analyzed via PMOD (version 3.6) and Inveon Research Workplace (version 4.2). For ex vivo biodistribution studies mice were euthanized 60 min p.i. and organs of interest were dissected, weighed, and measured in a γ-counter (PerkinElmer 2470 Wizard2, Perkin Elmer, Rodgau, Germany). The incorporated radioactivity was expressed as percentage of injected dose per gram of tissue (% ID/g).

## 7. Statistics

Errors in figures are given as standard deviation representing *n* = 3. Statistical analysis and calculation of IC_50_ was performed with graphpad prism software (version 5, GraphPad Software, La Jolla, CA, USA).

## Figures and Tables

**Table 1:** LogD values at pH 7.4 via shake-flask method.

Substance	logD_7.4_ Value
^18^F-DBCO	1.2 ± 0.07 [18]
^18^F-DBCO-folate	0.6 ± 0.07 [18]
^18^F-Alakyne	−1.18 ± 0.03
^18^F-Ala-folate	−1.43 ± 0.08

**Table 2:** k′ values at pH 2 via reversed-phase HPLC.

Substance	k′ Value	Retention Time (min)
native folic acid	0.3 [5]	3.07
^19^F-DBCO-folate	0.50 ± 0.10	3.26
^19^F-Ala-folate	0.27	2.89

**Table 3:** Overview IC_50_, pIC_50_ and K_i_ values for different folates using KB-cells and [^3^H]folic acid.

Substance	IC_50_ [nM]	pIC_50_	K_i_ [nM] ^1^
native folic acid	1.9	8.72	1.6 nM
^19^F-DBCO-folate	11.2 ± 3.7	7.98 ± 0.21	6.3 ±1.4
^19^F-Ala-folate	6.4 ± 0.5	8.20 ± 0.05	5.5 ± 0.4

^1^ according to Cheng-Prusoff equation with K_d_ ([^3^H]folic acid) = 1.0 nM.

**Table 4:** Overview of different tumor to organ ratios for ^18^F-DBCO-folate and ^18^F-Ala-folate.

Ratio	^18^F-DBCO-Folate	^18^F-Ala-Folate
tumor/blood	5.61	10.11
tumor/liver *	2.58	0.98
tumor/kidney	0.05	0.12
tumor/muscle	6.86	7.64

* including gallbladder.

## Supplementary Materials

The following are available online at http://www.mdpi.com/1424-8247/11/1/30/s1, all mass and NMR spectra, description of HPLC methods, Figure S1: Analytical radio-HPLC chromatogram of ^18^F-DBCO-folate, Figure S2: Analytical radio-HPLC chromatogram of ^18^F-Alakyne, Figure S3: Analytical radio-HPLC chromatogram of ^18^F-Ala-Folate, Figure S4: Stability of ^18^F-DBCO-folate and ^18^F-Ala-folate in human serum albumin at 37 °C for 1 and 2 h, Figure S5: FACS analysis of human KB and OC316 cells, Figure S6: Activity distribution of 5 nM ^18^F-DBCO-folate in uptake assay at 4 °C and 37 °C, Figure S7: Activity distribution of 5 nM ^18^F-Ala-folate in uptake assay at 4 °C and 37 °C, Table 1: Ex vivo biodistribution studies of ^18^F-DBCO-folate, Table 2: Ex vivo biodistribution studies of ^18^F-Ala-folate, Figure S8: Accumulation kinetics of ^18^F-DBCO-folate and ^18^F-Ala-Folate, Figure S9: MIP PET images of a KB-tumor bearing mice which received a blocking dose of folic acid.
