# Supplementary material for: Comparison Study of Two Differently Clicked 18F-Folates—Lipophilicity Plays a Key Role
Source: Pharmaceuticals (Basel). 2018 Mar 17;11(1):30. doi: 10.3390/ph11010030 (PMC5874726; doi:10.3390/ph11010030)
Supplement: Supplementary file 1 [file pharmaceuticals-11-00030-s001.pdf]

pharmaceuticals

Supplementary Materials

I. Analytics

Chemical shifts are reported in parts per million (ppm) relative to tetramethylsilane (0.00 ppm) for ^{1}H- and ^{13}C-NMR and trichloro-fluoro-methane (0.00 ppm) for ^{19}F-NMR. Coupling constants (J) are given in hertz (Hz) and the following abbreviations are used for the description of the NMR: singlet (s), doublet (d), triplet (t), quartet (q), multiplet (m), doublet of doublet (dd).

Semi preparative and analytical HPLC was performed with a Dinoex HPLC system equipped with a 680 HPLC pump and a UVD170U UV-detector (210 nm, 230 nm, 254 nm and 286nm) using a reversed-phase column (analytical column: Luna, C18, 5 µm, 250x4.6 mm; Gemini, C18, 5 µm, 250x4.6 mm; semi preparative column: Luna, C18, 5 µm, 250x20 mm; Synergi, C12, max-RP, 250x10 mm) at a flow rate of 3.5 mL/min for the semi preparative column and 1.0 mL/min for the analytical column unless otherwise stated. For radio-HPLC an additional GabiStar radiodetector (Raytest) was used. Dionex Chromeleon software was used for UV-data analysis and Raytest Gina star software for radioactivity detection.

Folate-azide

For the synthesis of the folate-azide, reported procedures from literature [12, 19] were slightly modified (see scheme S1.1 and S1.2). In brief, N-(tert-butoxycarbonyl)-L-glutamic acid-α-methyl-ester was coupled to 2-(2-(2-(2-azidoethoxy)ethoxy)ethoxy)ethan-1-amine using COMU as coupling agent and DIPEA to yield Boc-Glu(OMe)-PEG3-azide (*tert*-butyl-3-(2-(2-(2-(2-ethoxy)ethoxy)-ethoxy)ethylcarbamoylazid)-1-(methoxycarbonyl)-propylcarbamate). After deprotection, Glu(OMe) PEG3-azide was reacted with N^{2},N^{10}-diacetyl pteroic acid using COMU and DIPEA to give the final folate-azide.

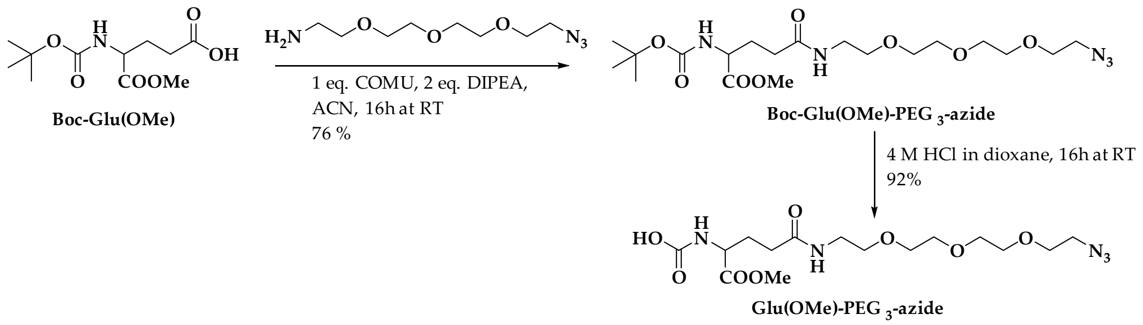

Scheme S1.1: Synthesis of Glu(OMe)-PEG3-azide (methyl-4-(2-(2-(2-(2-ethoxy)ethoxy)ethoxy)ethylcarbamoyl- azid)-3-aminobutanoate. Boc-Glu(OMe) = N-(tert-butoxycarbonyl)-L-glutamic acid-α- methylester; COMU = 1-cyano-2-ethoxy-2-oxoethylidenaminooxy)dimethylamino-morpholino- carbenium hexafluorophosphate; DIPEA = N,N-Diiso- propylethylamine;.

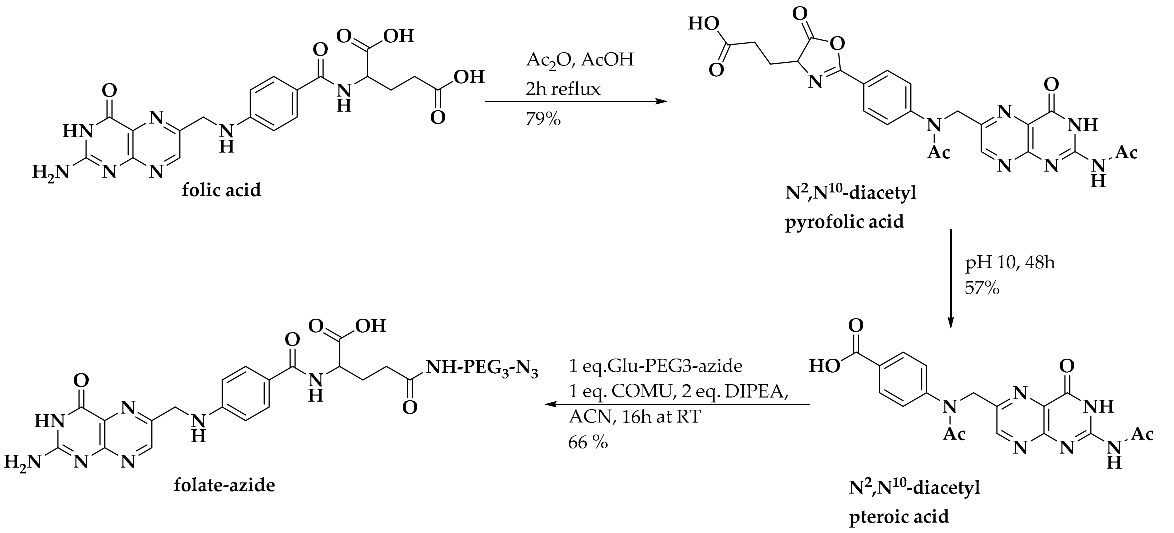

Scheme S1.2: Synthesis of folate-azide. Glu(OMe)-PEG3-azide (methyl-4-(2-(2-(2-(2-ethoxy)ethoxy)ethoxy)ethyl- carbamoyl- azid)-3-aminobutanoate; COMU = 1-cyano-2-ethoxy-2-oxoethylidenaminooxy)dimethylaminomorpholino-carbenium hexafluorophosphate; DIPEA = N,N-Diisopropylethylamine.

^{19}F-DBCO-folate

semi-preparative HPLC: solvent A is water, solvent B is acetonitrile; flow: 3.5 mL/min; method: 0-17 min 5-95% B, 17-19 min 95% B, 19-20 min 5-95% A

MS (ESI positive): m/z 562.3 ([M]$^{2+}$, 100%), 1123.3 ([M]$^{+}$, 10%), calculated for $C_{54}H_{66}FN_{13}O_{13}$: 1123.5.

Due to the low yields no NMR was recorded.

Analytical HPLC: solvent A is water with 0.1% TFA (trifluoroacetic acid) and solvent B is acetonitrile with 0.1% TFA. The following method was used: 0 - 40 min, 5 – 95% eluent B (gradient). Retention time is 21.89 min, purity ≥98%.

^{19}F-Ala-folate

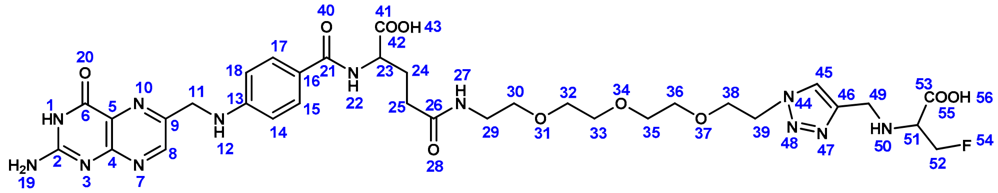

semi-preparative HPLC system: solvent A is water, solvent B is acetonitrile; flow: 3.6 mL/min; method: 0-5 min 0 % B, 5-14 min 0-60% B, 14-17 min 95% B ,17-18 min 95% B, 18-19 min 95-5% B.

Analytical HPLC: solvent A is ammonium formate solution (50 mM) and B is acetonitrile. The following method was used: 0-5 min 100% A (isocratic), 5-18 min 0-95% B (gradient), 18-22 min 95% B (isocratic), 22-25 min 5-100% A (gradient). Retention time 11.93 min, purity ≥97%.

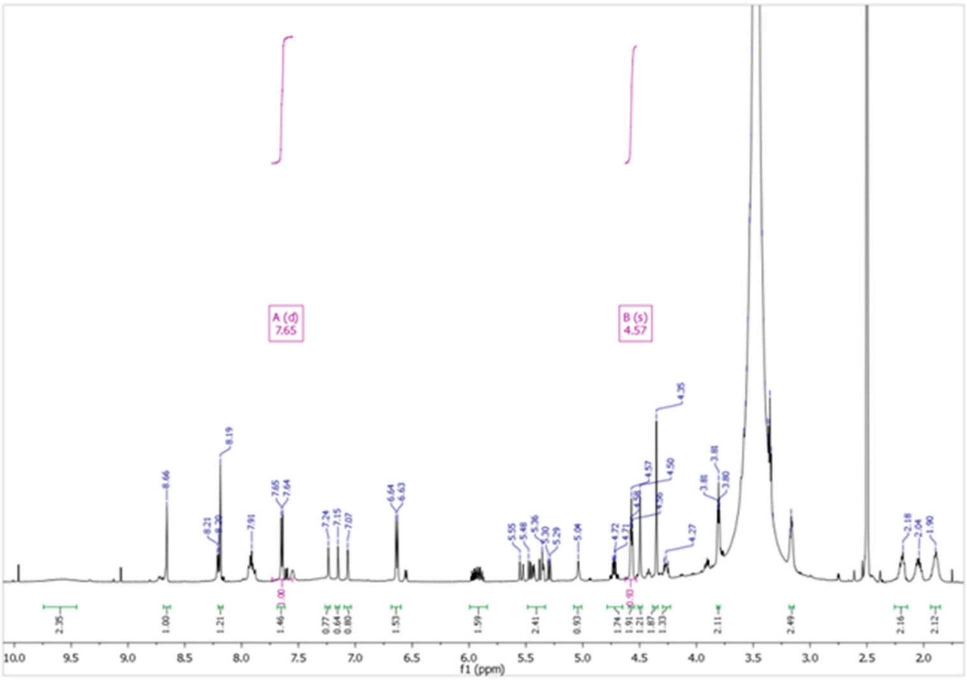

^{1}H-NMR (600 MHz, DMSO-d_6, Me_4Si): δ [ppm] = 1.90 – 2.04 (m, 2H, H-24), 2.19 (m, 2H, H-25), 3.34 – 3.58 (m, 13H, H-30 – H-38 and H-51), 3.80 – 3.81 (m, 2H, H-29), 4.25 – 4.28 (m, 1H, H-23), 4.35 (s, 2H, H-49), 4.50 (s, 1H, H-50), 5.04 (s, 1H, H-12), 4.56 – 4.58 (m, 2H, H-39), 4.71 – 4.74 (m, 2H, H-11), 5.36 – 5.48 (m, 2H,H-52), 6.64 (d, 2H, $^{3}J_{H\text{-}H}$ = 8.5 Hz, H-14/18), 7.07 (s, 1H, H-27), 7.15 (s, 1H, H-1), 7.24 (s, 1H, H-22), 7.65 (d,2H, $^{3}J_{H\text{-}H}$ = 8.5 Hz, H-15/17), 8.20 (s, 1H, H-45), 8.67 (s, 1H, H-8), 9.57 (br, 2H, H-19).

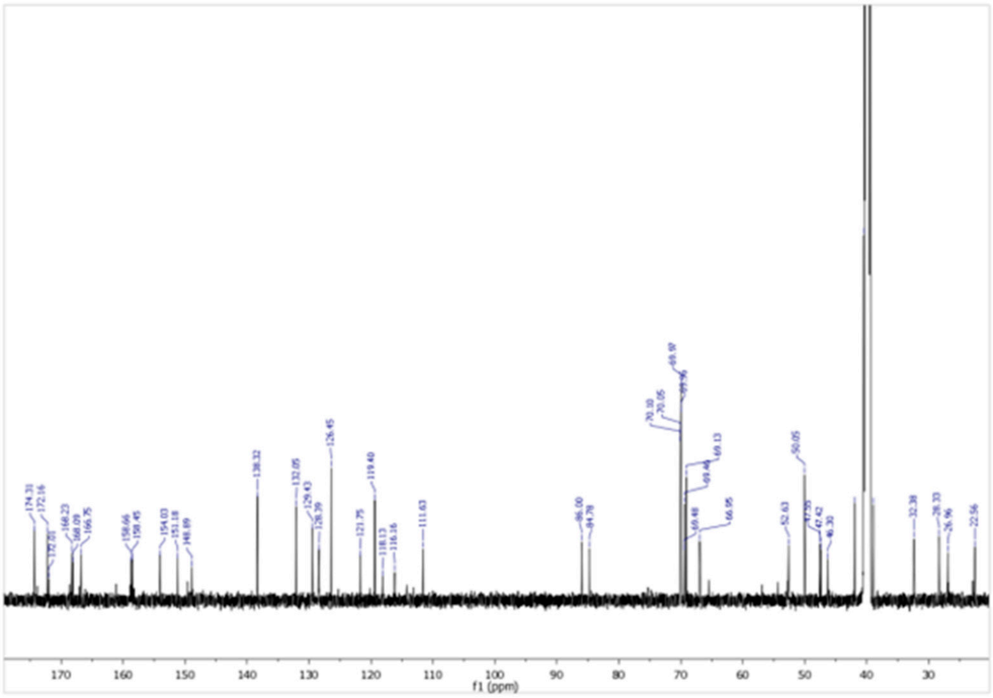

^{13}C-NMR (600 MHz, DMSO-d_6) δ [ppm] = 26.9 & 27.1 (C24), 32.3 (C25), 42.0 (C49), 47.3 (C51), 50.3 (C39), 52.7 (C23), 66.9 (C11), 69.1 (C29), 69.5 – 70.1 (C30 – C38), 111.6 (C14/18), 126.5 (C45), 128.5 (C16), 129.4 (C15/17), 132.1 (C9), 138.3 (C46), 148.9 (C8), 151.2 (C13), 154.1 (C4), 158.5 (C2/5), 158.7 (C2/5), 166.8 (C21), 168.1 (C6/53), 168.2 (C6/53), 172.3 (C26), 174.1 (C41).

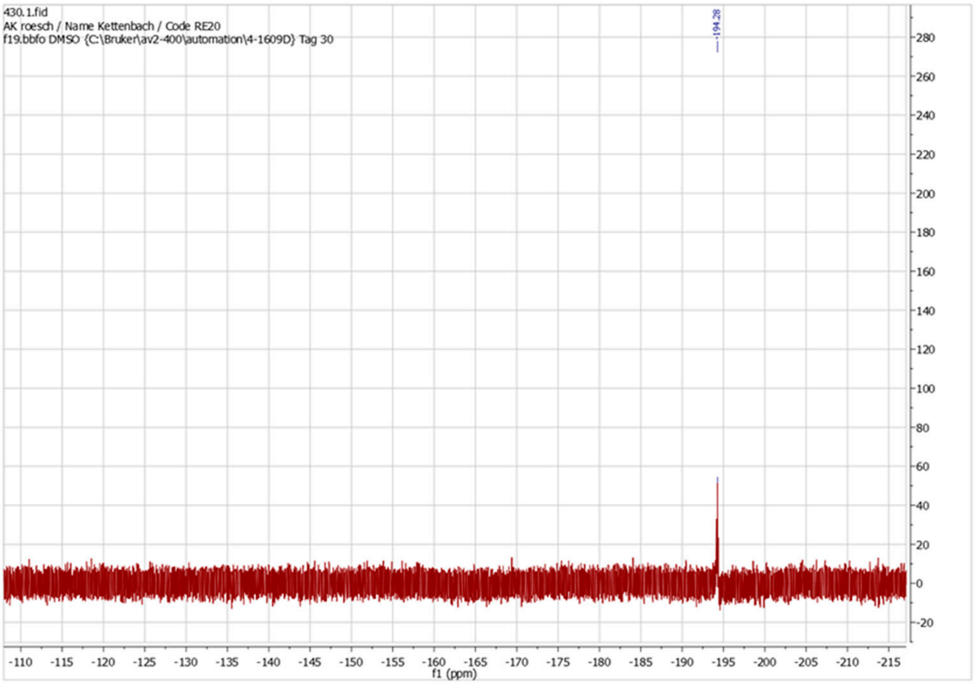

^{19}F-NMR (400 MHz, DMSO-d_6) δ [ppm] = -194.3 (F-54)

MS (ESI positive): m/z (% rel Int): 394.2 ($[M]^{2+}$, 100%), 787.3 ($[M]^{+}$, 80%), calculated for $C_{33}H_{43}FN_{12}O_{10}$: 786.32.

II. ^{18}F-Radiolabeling

II.1. General radiolabeling methods

Radiosyntheses were performed manually (starting activities ≤ 8 GBq) or in a manipulator-equipped hot cell (starting activities > 8GBq) using conventional heating. N.c.a. [^{18}F]fluoride was produced using the ^{18}O(p,n)^{18}F nuclear reaction. The aqueous ^{18}F-solution was trapped on an Sep Pak light Waters Accell Plus QMA cartridge, which was pre-conditioned with 1.0M potassium carbonate solution (10 mL) and millipore water (10 mL). For elution, a solution of Kryptofix© (5 mg, 13 µmol) and potassium carbonate (1 mg, 7.5 µmol) in 600 µL (acetonitrile:water/1:1) was used. The azeotropic drying was performed at 85 °C for 20 min under reduced pressure (250 mbar) and helium flow. Within this time, dry acetonitrile (3 x 1.0 mL) was added and evaporated to yield the final dry [^{18}F]fluoride-base mixture.

II.3. ^{18}F-DBCO-folate

semi-preparative HPLC: solvent A is water, solvent B is acetonitrile; flow of 2.5 mL/min, isocratic method: 50% A

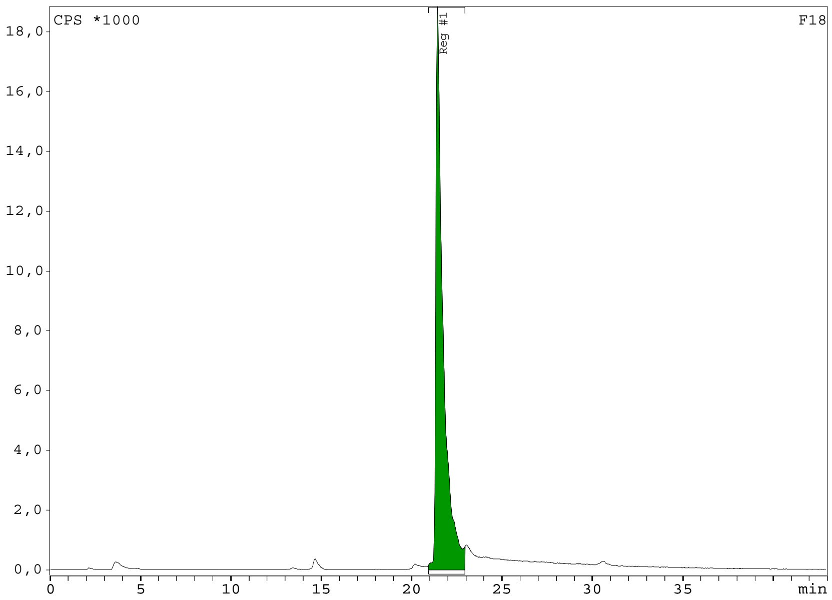

Figure S1. Analytical radio-HPLC chromatogram of ^{18}F-DBCO-folate. Analytical radio-HPLC was performed using water with 0.1% trifluoroacetic acid (TFA) as eluent A and eluent B was acetonitrile with 0.1% TFA. The following gradient method was used: 0 - 40 min, 5 – 95% B.

II.2. Synthesis of ^{18}F-Alakyne

semi-preparative HPLC: solvent A is 50 mM ammonium formate solution, solvent B is acetonitrile; flow of 3.6 mL/min, gradient method: 0-5 min 100% A , 5-18 min 0-95% B, 18-22 min 95% B, 22-25 min 5-100% A

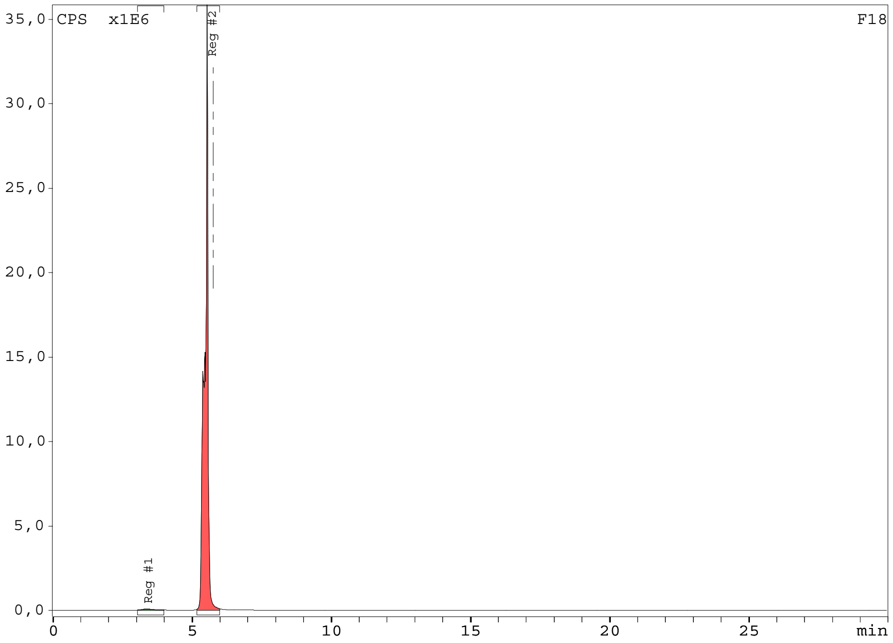

Figure S2. Analytical radio-HPLC chromatogram of ^{18}F-Alakyne. Analytical radio-HPLC was performed with the same gradient as described for the semi-preparative HPLC.

II.3. ^{18}F-Ala-Folate

semi-preparative HPLC: see ^{18}F-Alakyne

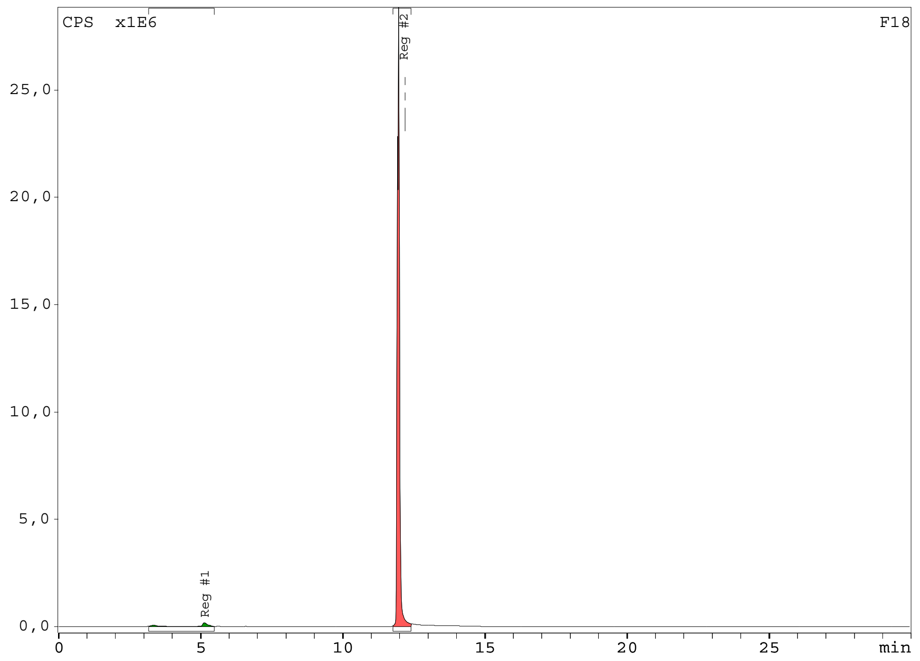

Figure S3. Analytical radio-HPLC chromatogram of ^{18}F-Ala-Folate. Analytical radio-HPLC was performed with the same gradient method as described for the semi-preparative HPLC.

III. *In vitro* studies

III.1. Stability in human serum albumin

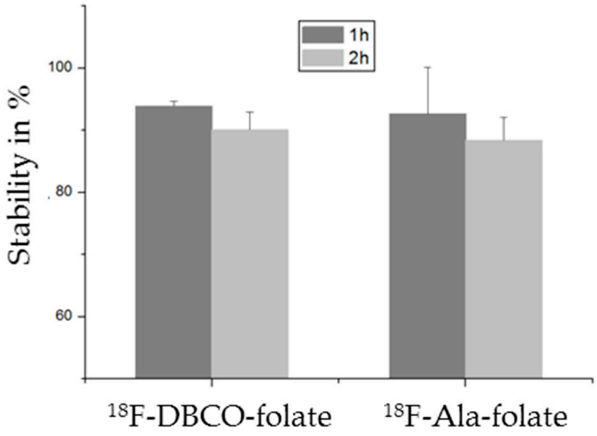

Figure S4. Stability of ^{18}F-DBCO-folate and ^{18}F-Ala-folate in human serum albumin at 37 °C for 1h and 2h.

III.2. FACS analysis of human KB and OC316 cells

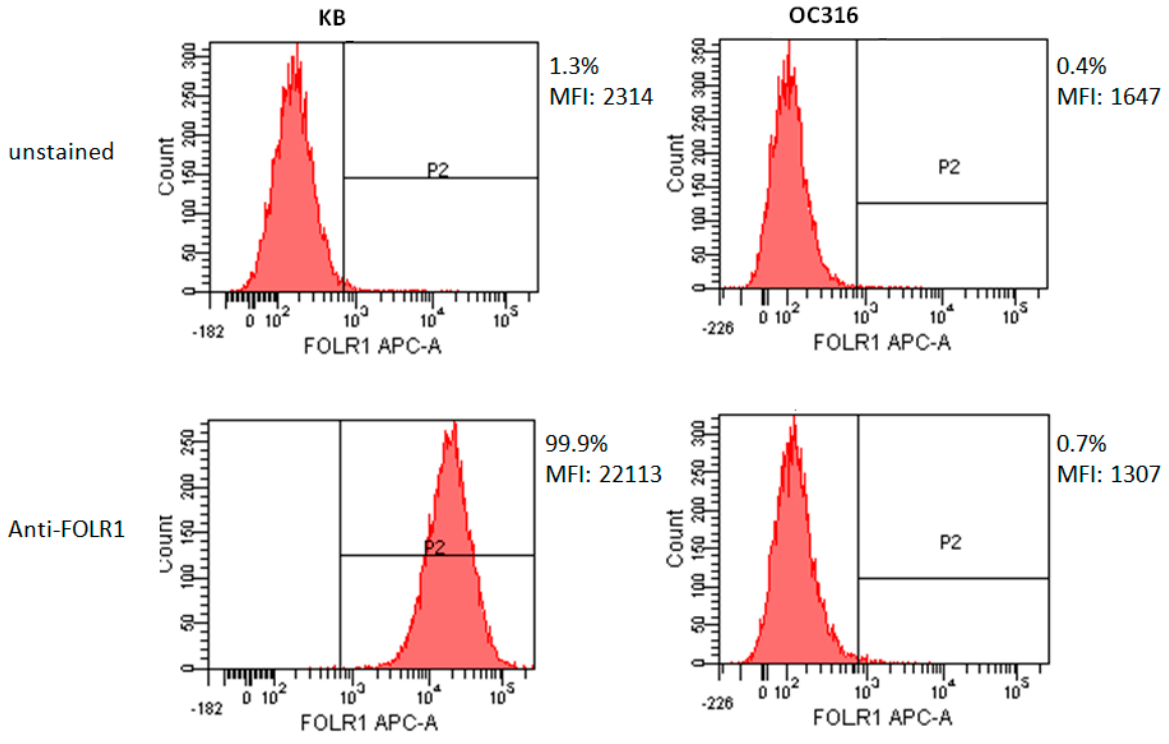

Figure S5. FACS analysis of human KB and OC316 cells. 1x10[6] cells were stained with 0.25 µg of anti-human FOLR1-APC (R&D System) and analyzed by a LSRII (Becton Dickinson) flow cytometer equipped with DIVA software (version 6.0).

III.3. PIE-charts for uptake assay

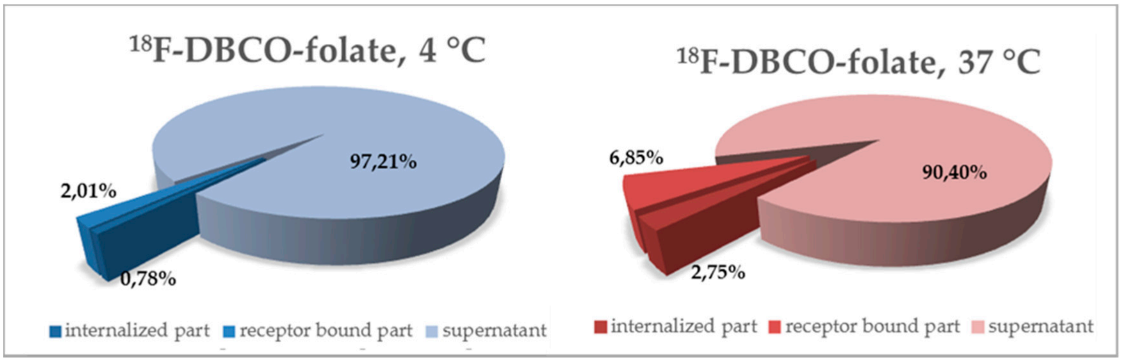

Figure S6. Activity distribution of 5 nM ^{18}F-DBCO-folate in uptake assay at 4 °C (a) and 37 °C (b).

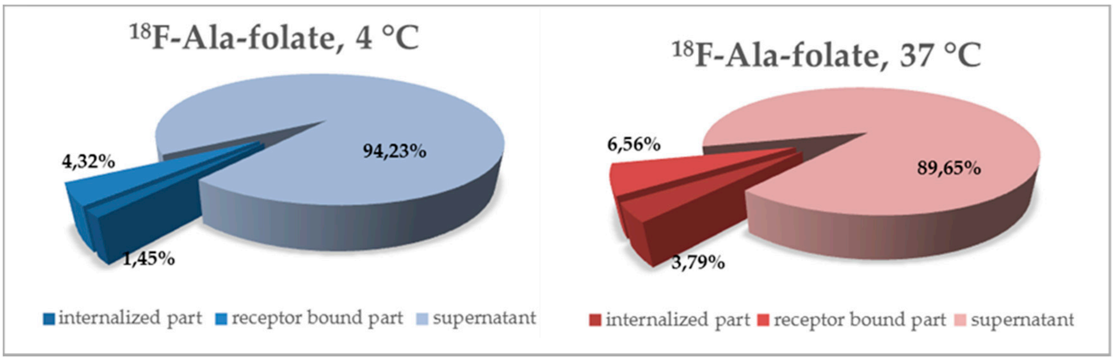

Figure S7. Activity distribution of 5 nM ^{18}F-Ala-folate in uptake assay at 4 °C (a) and 37 °C (b).

IV. *Ex vivo* biodistribution

Table 1. *Ex vivo* biodistribution studies of ^{18}F-DBCO-folate in healthy and KB tumor bearing balb/c and balb/c nu/nu mice after 60 min p.i. Errors are given as standard deviation. Bold framed cells reflect FR-positive tissues. n.d. = no data.

	**healthy balb/c mice**		**balb/c mice, KB xenograft**	
	**60 min p.i.**	**60 min p.i.**	**60 min p.i.**	**60 min p.i.**
	**(n = 4)**	**Blocked[a] (n = 5)**	**(n = 4)**	**Blockade[a] (n = 5)**
**organ/tissue**	**[%ID/g tissue]**	**[%ID/g tissue]**	**[%ID/g tissue]**	**[%ID/g tissue]**
Pancreas	0.08 ± 0.01	0.09 ± 0.05	0.07 ±0.01	0.03 ± 0.02
Inguinal lymph nodes	0.18 ± 0.08	0.10 ± 0.08	0.48 ± 0.14	0.04 ± 0.02
Lung	0.35 ± 0.27	0.36 ± 0.11	0.17 ±0.06	0.13 ± 0.05
Blood	0.08 ± 0.01	0.12 ± 0.05	0.09 ± 0.04	0.06 ± 0.04
Heart	0.09 ± 0.01	0.11 ± 0.06	0.08 ± 0.02	0.04 ± 0.02
Liver	0.24 ± 0.02	0.33 ± 0.08	0.18 ± 0.07	0.14 ± 0.06
Intestines (empty)	0.87 ± 0.57	0.33 ± 0.22	0.42 ± 0.52	0.56 ± 0.81
Spleen	0.07 ± 0.02	0.11 ± 0.06	0.06 ± 0.02	0.05 ± 0.03
Left kidney	3.90 ± 0.38	0.40 ± 0.07	4.83 ± 0.90	0.30 ± 0.04
Right kidney	3.99 ± 0.37	0.38 ± 0.07	4.76 ± 0.89	0.30 ± 0.02
Muscle	0.06 ± 0.01	0.07 ± 0.05	0.07 ± 0.01	0.02 ± 0.01
Stomach (empty)	n.d.	n.d.	0.82 ± 0.64	0.22 ± 0.09
Appendix	n.d.	n.d.	0.13 ± 0.02	0.07 ± 0.01
**Tumor**			**0.48 ± 0.14**	**0.09 ± 0.04**

[a]In the blocking group, each animal received 100 µg/100 µL of native folic acid in phosphate buffered saline (PBS) 2 min before radiotracer injection.

Table 2. *Ex vivo* biodistribution studies of ^{18}F-Ala-folate in healthy and KB tumor bearing balb/c and balb/c nu/nu mice after 60 min p.i. Errors are given as standard deviation. Bold framed cells reflect FR-positive tissues. n.d. = no data.

	**healthy balb/c mice**		**balb/c mice, KB xenograft**	
	**60 min p.i.**	**60 min p.i.**	**60 min p.i.**	**60 min p.i.**
	**(n = 3)**	**Blocked[a] (n = 3)**	**(n = 5)**	**Blockade[a] (n = 4)**
**organ/tissue**	**[%ID/g tissue]**	**[%ID/g tissue]**	**[%ID/g tissue]**	**[%ID/g tissue]**
Pancreas	0.33 ± 0.20	0.16 ± 0.04	0.26 ± 0.11	0.12 ± 0.08
Inguinal lymph nodes	0.93 ± 0.67	0.27 ± 0.15	0.57 ± 0.15	0.17 ± 0.12
Lung	0.36 ± 0.16	0.38 ± 0.05	0.28 ± 0.05	0.19 ± 0.10
Blood	0.29 ± 0.29	0.18 ± 0.01	0.17 ± 0.05	0.13 ± 0.06
Heart	0.32 ± 0.21	0.14 ± 0.04	0.22 ± 0.09	0.09 ± 0.05
Liver	1.51 ± 1.26	1.63 ± 0.32	1.71 ± 1.02	1.34 ± 0.67
Intestines (empty)	1.67 ± 1.12	4.86 ± 3.97	3.42 ± 2.18	1.49 ± 1.23
Spleen	0.15 ± 0.09	0.14 ± 0.03	0.16 ± 0.04	0.11 ± 0.06
Left kidney	19.90 ± 8.63	1.88 ± 0.44	14.49 ± 3.42	1.07 ± 0.49
Right kidney	20.55 ± 9.71	1.72 ± 0.58	14.27 ± 3.35	1.00 ± 0.36
Muscle	0.26 ± 0.13	0.15 ± 0.02	0.22 ± 0.02	0.22 ± 0.13
Stomach (empty)	0.71 ± 0.24	1.99 ± 1.68	0.95 ± 0.50	2.50 ± 1.80
Appendix	0.20 ± 0.11	3.11 ± 1.71	0.39 ± 0.22	0.13 ± 0.04
**Tumor**			**1.68 ± 0.13**	**0.26 ± 0.06**

[a]In the blocking group, each animal received 100 μg/100 μL of folic acid in phosphate buffered saline (PBS) 2 min before radiotracer injection.

V. Time-Activity Curve

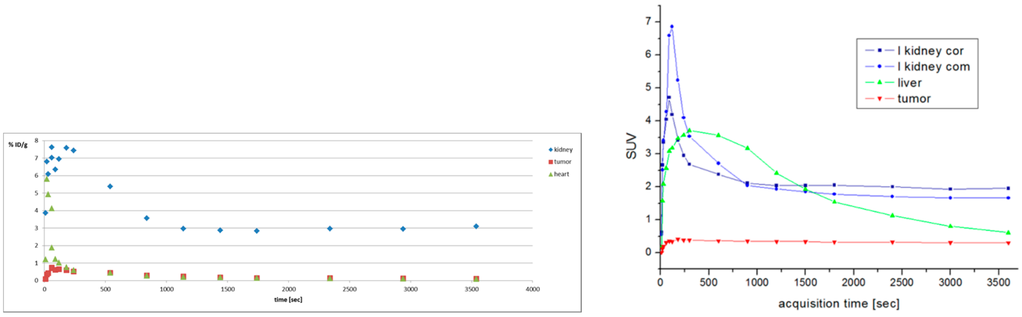

Figure S8: Accumulation kinetics of ^{18}F-DBCO-folate and ^{18}F-Ala-Folate. Analysis of a dynamic PET scan over 60 min p.i.

VI. PET/MR-studies

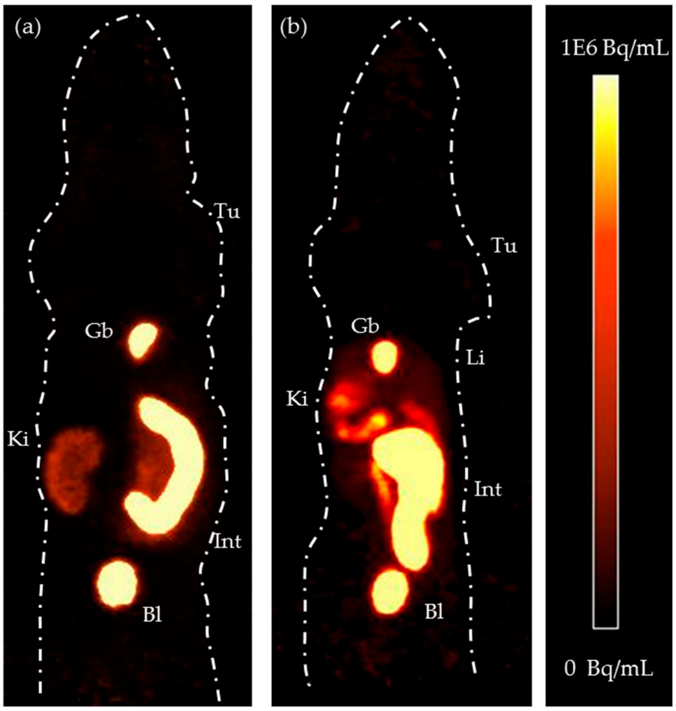

Figure S9: MIP PET images of a KB-tumor bearing mouse which received blocking dose of folic acid. Static scan over 10 min 50 min p.i. (a) ^{18}F-DBCO-folate and (b) ^{18}F-Ala-folate. Tu = KB-tumor, Gb = gallbladder, Li = liver, Ki = kidney, Int = intestines, Bl = bladder.
